# Knowledge, attitude, and practice of healthcare professionals toward cognitive dysfunction in Parkinson’s disease and cognitive rehabilitation

**DOI:** 10.1186/s12909-023-04989-5

**Published:** 2024-01-04

**Authors:** Xia Cai, Fei Chen, Shufang Wang, Pinglei Pan, Tianchi Mu, Congsong Dong, Zhenyu Dai, Zhipeng Chen

**Affiliations:** 1 Department of Public Health Management and Preventive Care, The Yancheng School of Clinical Medicine of Nanjing Medical University (Yancheng Third People’s Hospital), 224008 Yancheng, China; 2 Department of Academic Research, The Yancheng School of Clinical Medicine of Nanjing Medical University (Yancheng Third People’s Hospital), 224008 Yancheng, China; 3 Department of Radiology, The Yancheng School of Clinical Medicine of Nanjing Medical University (Yancheng Third People’s Hospital), 224008 Yancheng, China; 4 Department of Neurology, The Yancheng School of Clinical Medicine of Nanjing Medical University (Yancheng Third People’s Hospital), 224008 Yancheng, China

**Keywords:** Parkinson’s Disease, Healthcare professionals, Knowledge, Attitude, Practice

## Abstract

**Background:**

To investigate the knowledge, attitude, and practice (KAP) of healthcare professionals regarding cognitive dysfunction and cognitive rehabilitation in Parkinson’s disease (PD).

**Methods:**

This multicenter, cross-sectional survey enrolled physicians and nurses in 10 hospitals between October 2022 and November 2022. A self-administered questionnaire was developed to collect the demographic information of the participants and their knowledge, attitude, and practice toward cognitive dysfunction in PD and cognitive rehabilitation.

**Results:**

This study enrolled 224 physicians and 229 nurses. The knowledge, attitude, and practice scores were 12.57 ± 3.76 (total score: 22), 29.10 ± 3.71 (total score: 32), and 21.07 ± 8.03 (total score: 28) among physicians, and 9.97 ± 4.70 (total score: 22), 25.27 ± 8.96 (total score: 32), and 25.27 ± 8.96 (total score: 28) among nurses. Among physicians, the knowledge scores (OR = 4.23, 95%CI: 2.36–7.58, *P* < 0.001) and attitude scores (OR = 3.00, 95%CI: 1.67–5.37, *P* < 0.001) were independently associated with good practice. Among nurses, the knowledge scores (OR = 4.31, 95%CI: 2.31–8.05, *P* < 0.001), attitude scores (OR = 5.18, 95%CI: 2.82–9.53, *P* < 0.001), working department (Ref: rehabilitation; neurology: OR = 2.26, 95%CI: 1.01–5.08, *P* = 0.048; public health service/chronic disease follow-up center: OR = 2.98, 95%CI: 1.12–7.92, *P* = 0.028) were independently associated with good practice.

**Conclusions:**

Physicians and nurses have insufficient knowledge, favorable attitudes, and active practice regarding cognitive dysfunction and cognitive rehabilitation in PD. This study identified gaps in KAP and suggested education activities to improve the KAP toward cognitive dysfunction in PD.

**Supplementary Information:**

The online version contains supplementary material available at 10.1186/s12909-023-04989-5.

## Background

Parkinson’s disease (PD) is a progressive movement disorder characterized by bradykinesia, resting tremor, muscular rigidity, and the loss of postural reflexes [1]. The prevalence of PD increases with age (1% in patients > 65 years old and 3% in patients > 80 years old) [2]. PD is due to a loss of dopaminergic neurons in the substantia nigra and other dopaminergic and nondopaminergic areas of the brain [3–5]. PD is related to cognitive complications, including dementia, anxiety, depression, sleep disorders, and psychosis [6–10]. The overall treatment for PD is individualized and aims at reducing movement dysfunction, tremor, and postural instability while managing cognitive changes and minimizing side effects [2, 5]. The management of cognitive impairment includes lifestyle modification, counseling, coaching, and rivastigmine [11, 12]. Cognitive rehabilitation is a promising treatment for cognitive impairment in PD [13–15].

Still, cognitive rehabilitation for PD is a novel approach, and the knowledge, attitudes, and practices (KAP) of healthcare providers towards it are unknown. Cognitive rehabilitation for PD requires a specific set of knowledge and skills that can enable a healthcare professional to perform cognitive rehabilitation adequately or at least refer the patient to a qualified professional. Each patient is unique and suffers from a unique disease, and a proper knowledge of cognitive rehabilitation for PD is necessary for individualized therapy. KAP surveys are designed to provide quantitative and qualitative assessments of specific individuals towards a specific subject/activity. It is useful to identify the gaps in KAP that should be targeted in teaching, training, and continuous education [16, 17]. Still, recent studies identified important gaps in knowledge and practice among physicians regarding managing PD [18, 19], but they did not specifically focus on cognitive dysfunction and cognitive rehabilitation.

Therefore, this study aimed to investigate the knowledge, attitude, and practice of healthcare professionals regarding cognitive dysfunction in PD and cognitive rehabilitation.

## Methods

### Study design and participants

This multicenter, cross-sectional survey enrolled physicians and nurses in 10 hospitals in Jiangsu, China, between October 25, 2022, and November 10, 2022. The inclusion criteria were (1) certified physicians or nurses and (2) working in the Department of Neurology, Rehabilitation, Public Health Service, or Chronic Disease Follow-up Centers. This work has been carried out in accordance with the Declaration of Helsinki (2000) of the World Medical Association. The study was approved by the Medical Ethics Committee of Yancheng Third People’s Hospital (approval No. 2022-72). Written informed consent was provided by all participants before the survey.

### Questionnaire and data collection

The questionnaire was designed by reviewing the published literature and referring to the Diagnostic Criteria and Treatment Guidelines for Parkinson’s Disease Dementia [20], the Recommendations of Chinese Parkinson’s Disease and Movement Disorder Society Consensus on Therapeutic Management of Parkinson’s Disease (4th edition) [21], the Chinese Guidelines for the Diagnosis and Treatment of Mild Cognitive Impairment in Parkinson’s Disease (2020) [22], and the Diagnostic Criteria for Mild Cognitive Impairment in Parkinson’s Disease: Movement Disorders Society Task Force Guidelines [23]. The first draft of the questionnaire was revised according to the comments made by two experts (one expert neurologist and one expert in neurorehabilitation, both with > 20 years of experience). Sixty-two questionnaires were distributed for the pre-test, showing a Cronbach’s α of 0.919 and a Kaiser-Meyer-Olkin (KMO) of 0.814.

The final questionnaire was in Chinese (an English translation is provided as Supplementary Material) and included four sections with 35 items. Among them, eight items were about demographic information, 12 were in the knowledge section, eight were in the attitude section, and seven were in the practice section. In the knowledge section, the questions with correct answers were scored 2 for each correct answer and 0 for wrong or uncertain answers; a correct statement with “Have known”, “Know a little”, and “Don’t know” were scored 2, 1, and 0, respectively. One trap question (K5) was set to eliminate illogical answers from the questionnaire and was not counted in the score. Thus, the knowledge scores ranged from 0 to 22. The attitude and practice section were scored using a 5-point Likert scale, ranging from very positive (4 points) to very negative (0 points). The total scores for the attitude ranged from 0 to 32, while the total scores for the practice ranged from 0 to 28.

The questionnaire was distributed to participants through the Sojump website (https://www.wjx.cn). All questions were mandatory for questionnaire submission. Questionnaires with missing answers, an obvious filling pattern (e.g., all last choices), or an error in the trap questions (K5 and K12; K5 was not included in the analyses) were excluded. Only one questionnaire could be submitted for each IP address.

### Statistical analysis

STATA 17.0 (STATA Corp., College Station, TX, USA) was used for statistical analysis. The continuous variables were presented as the arithmetic means ± standard deviations (without any weight). The continuous variables with a normal distribution were tested using Student’s t-test or analysis of variance (ANOVA), and those with a skewed distribution using the Wilcoxon-Mann-Whitney test or Kruskal-Wallis analysis of variance. Categorical data were presented as n (%) and analyzed using the chi-square test. Pearson’s correlation was used for correlation analysis. The 70th percentile of the knowledge, attitude, and practice scores was used as a cut-off in logistic regression, and the participants were dichotomized as low score (< 70%) or high score (≥ 70%). Logistic regression was performed using low/high scores as the dependent variable. The variables with significant differences in univariable logistic regression analyses were included in the multivariable logistic regression analyses. A confirmatory factor analysis (CFA) was performed to determine the fit of the KAP model. Two-sided *P* < 0.05 were considered statistically significant.

## Results

A total of 230 physicians and 238 nurses from 10 hospitals participated in this study. Five questionnaires from physicians and 22 from nurses were excluded due to missing answers, an obvious filling pattern, or an error in the trap question. Therefore, 225 valid questionnaires from physicians and 216 from nurses were included in this study. Among them, most physicians were female (58.04%), 31–40 years of age (44.54%), had junior college/bachelor’s degree education (65.62%), were working in tertiary hospitals (67.41%), were working in neurology department (50.00%), had a middle title (32.14%), and had ≥ 10 years of working experience (39.73%) (Table 1). Most nurses were female (97.38%), 31–40 years of age (44.54%), had junior college/bachelor’s degree education (96.51%), were working in tertiary hospitals (59.83%) and neurology department (58.52%), with a primary title (41.92%) and ≥ 10 years of working experience (51.97%) (Table 2).

**Table 1:** Characteristics of the physicians

Characteristics	n (%)	Knowledge Score		Attitude Score		Practice Score	
		Mean ± SD	***P***	Mean ± SD	***P***	Mean ± SD	***P***
**Physician**	224	12.57 ± 3.76		29.10 ± 3.71		23.39 ± 4.41	
**Gender**			0.380		0.406		0.181
Male	94 (41.96)	12.80 ± 3.85		29.29 ± 3.73		23.78 ± 4.49	
Female	130 (58.04)	12.40 ± 3.69		28.96 ± 3.71		23.12 ± 4.34	
**Age, years**			0.383		0.099		0.288
≤ 30	80 (34.93)	12.34 ± 3.96		29.30 ± 3.40		23.08 ± 4.42	
31–40	102 (44.54)	12.45 ± 3.67		28.50 ± 4.20		23.40 ± 4.15	
≥ 41	47 (20.52)	13.29 ± 3.58		30.17 ± 2.66		23.98 ± 5.01	
**Education**			0.267		0.765		0.387
Junior college/bachelor’s degree	147 (65.62)	12.41 ± 3.63		29.12 ± 3.79		23.20 ± 4.47	
Master’s degree and above	77 (34.38)	12.87 ± 4.00		29.05 ± 3.58		23.75 ± 4.31	
**Institution**			0.544		0.611		0.755
Public primary / secondary hospital	63 (28.12)	12.11 ± 4.02		29.46 ± 3.42		23.14 ± 4.35	
Public tertiary hospital	151 (67.41)	12.73 ± 3.63		28.96 ± 3.84		23.52 ± 4.39	
Private hospital	10 (4.46)	13.00 ± 4.14		28.90 ± 3.75		23.10 ± 5.40	
**Department**			0.555		0.482		0.221
Neurology	112 (50.00)	12.71 ± 3.68		29.18 ± 3.76		23.22 ± 4.74	
Rehabilitation	83 (37.05)	12.22 ± 3.85		29.14 ± 3.81		23.99 ± 3.92	
Public health service / chronic disease follow-up center	29 (12.95)	13.00 ± 3.85		28.66 ± 3.32		22.34 ± 4.28	
**Professional title**			0.053		0.589		0.336
None	27 (12.05)	12.44 ± 3.13		29.15 ± 2.86		23.30 ± 4.28	
Primary	66 (29.46)	12.39 ± 4.11		29.30 ± 3.52		22.74 ± 4.52	
Intermediate	72 (32.14)	11.88 ± 3.85		28.50 ± 4.57		23.49 ± 4.21	
Vice-senior / Senior	59 (26.34)	13.66 ± 3.32		29.58 ± 3.02		24.05 ± 4.59	
**Years of work**			0.124		0.796		0.136
< 5 years	78 (34.82)	12.24 ± 4.12		29.05 ± 3.46		23.10 ± 4.41	
5-9.9 years	57 (25.45)	11.93 ± 4.00		28.84 ± 4.09		22.74 ± 4.51	
≥ 10 years	89 (39.73)	13.26 ± 3.15		19.30 ± 3.71		24.07 ± 4.30	

**Table 2:** Demographic characteristics of the nurses

Characteristics	n (%)	Knowledge Score		Attitude Score		Practice Score	
		Mean ± SD	***P***	Mean ± SD	***P***	Mean ± SD	***P***
**Nurse**	229	9.97 ± 4.70		25.27 ± 8.96		21.07 ± 8.03	
**Sex**			0.037		0.561		0.085
Male	6 (2.62)	5.67 ± 5.24		19.33 ± 15.27		14.33 ± 11.67	
Female	223 (97.38)	10.09 ± 4.64		25.43 ± 8.73		21.26 ± 7.87	
**Age, years**			0.202		0.108		0.808
≤ 30	91 (39.74)	9.59 ± 5.06		24.74 ± 9.96		20.75 ± 8.78	
31–40	102 (44.54)	10.75 ± 4.00		26.69 ± 6.90		21.84 ± 6.59	
≥ 41	36 (15.72)	8.72 ± 5.30		22.61 ± 10.79		19.72 ± 9.62	
**Education**			0.508		0.035		0.397
Junior college/bachelor’s degree	221 (96.51)	9.91 ± 4.71		25.10 ± 9.06		20.97 ± 8.12	
Master’s degree and above	8 (3.49)	11.63 ± 4.57		30.13 ± 2.95		23.88 ± 4.58	
**Institution**			0.238		0.422		0.149
Public primary/secondary hospital	75 (32.75)	9.75 ± 4.32		25.51 ± 7.97		21.52 ± 7.06	
Public tertiary hospital	137 (59.83)	10.37 ± 4.76		25.61 ± 8.88		21.39 ± 8.08	
Private hospital	17 (7.42)	7.71 ± 5.35		21.47 ± 12.78		16.59 ± 10.51	
**Department**			0.029		0.142		0.009
Neurology	134 (58.52)	10.80 ± 3.75		27.13 ± 5.32		22.69 ± 5.48	
Rehabilitation	48 (20.96)	8.00 ± 5.88		20.77 ± 12.83		16.50 ± 10.83	
Public health service/chronic disease follow-up center	47 (20.52)	9.62 ± 5.23		24.55 ± 10.78		21.15 ± 9.16	
**Professional title**			0.342		0.949		0.078
None	19 (8.30)	8.63 ± 4.81		24.05 ± 10.99		17.47 ± 9.08	
Junior	96 (41.92)	9.82 ± 4.83		25.35 ± 9.17		21.51 ± 8.18	
Intermediate	80 (34.93)	10.56 ± 4.58		25.43 ± 8.39		21.00 ± 7.63	
Vice-senior / Senior	34 (14.85)	9.74 ± 4.57		25.35 ± 8.80		22.03 ± 7.72	
**Years of work**			0.361		0.505		0.714
< 5 years	62 (27.07)	9.19 ± 4.74		25.11 ± 10.28		19.98 ± 9.08	
[5, 10) years	48 (20.96)	10.44 ± 5.12		24.85 ± 8.83		21.40 ± 7.70	
≥ 10 years	119 (51.97)	10.18 ± 4.49		25.52 ± 8.32		21.51 ± 7.58	

The mean knowledge score of the physicians was 12.57 ± 3.76 (total score: 0–22, 57.14%), indicating insufficient knowledge. Among all knowledge items, only K5 (“MCI in PD might appear in the early stage of the disease and may even precede the onset of motor impairment”) was correctly answered by more than 60% of the physicians (Table 3). Among the nurses, the mean knowledge score was 9.97 ± 4.70 (total score: 0–22, 45.32%), which was insufficient. The items K5 (“MCI in PD might appear in the early stage of the disease and may even precede the onset of motor impairment”) and K2 (“MCI is an intermediate state between normal cognitive function and PDD”) were correctly answered by more than 60% nurses (Table 3). The knowledge scores varied among nurses of different genders (*P* = 0.037) and working departments (*P* = 0.029) (Table 2).

**Table 3:** Knowledge regarding cognitive dysfunction in Parkinson’s disease and cognitive rehabilitation

Knowledge	Known/correct n (%)	
	Physicians	Nurses
K1. Cognitive dysfunction is one of the common non-motor symptoms of PD, including mild cognitive impairment (MCI) and Parkinson’s disease dementia (PDD).	23 (10.27)	17 (8.13)
K2. MCI is an intermediate state between normal cognitive function and PDD.	105 (46.88)	139 (66.51)
K3. The risk factors for the development of dementia in PD patients.	107 (47.77)	72 (34.45)
K4. The medications that should be discontinued in PD patients with Cognitive dysfunction.	40 (17.86)	25 (11.96)
K5. MCI in PD can appear in the early stage of the disease and may even precede the onset of motor impairment.	193 (86.16)	165 (78.95)
K6. The Montreal Cognitive Assessment (MoCA), the Parkinson′s Disease Cognitive Rating Scale (PD-CRS), and the Mattis Dementia Rating Scale-2 (MDRS-2) are the three scales currently recommended for evaluating overall cognitive function in PD with better validity and reliability.	72 (32.14)	27 (12.92)
K7. The rehabilitation treatments for PD have proven effective.	103 (45.98)	50 (23.92)
K8. How to carry out cognitive training for patients.	80 (35.71)	46 (22.01)
K9. Aerobic exercise may be effective in improving executive function in PD patients, and appropriate aerobic exercise, such as horizontal exercise bicycles, is recommended for PD patients with MCI.	92 (41.07)	50 (23.92)
K10. Transcranial direct current stimulation (tDCS) treatment may be considered for PD patients with MCI.	88 (39.29)	45 (21.53)
K11. The dietary precautions for PD patients.	103 (45.98)	65 (31.10)

The mean attitude score was 29.10 ± 3.71 (total score: 0–32, 90.94%) in the physicians and 25.27 ± 8.96 (78.97%) in the nurses, indicating a favorable attitude (Tables 1 and 2). More than 50% of the physicians and more than 40% of the nurses agreed that “some physicians and nurses lack awareness and attention to cognitive impairment in PD and its rehabilitation interventions”. The physicians had higher scores than the nurses for items A2, A3, A5, A6, A7, and A8 (all *P* < 0.050) (Table 4).

**Table 4:** Attitude regarding cognitive dysfunction in Parkinson’s disease and cognitive rehabilitation

Attitude	Physicians, n (%)	Nurses, n (%)	***P***
	Mean ± SD	Mean ± SD	
A1. Early diagnosis and intervention of MCI in patients with PD are of clinical importance.	3.74 ± 0.59	3.68 ± 0.61	0.192
A2. The individualized rehabilitation intervention program for patients with PD needs to be developed based on the patient’s condition.	3.75 ± 0.52	3.64 ± 0.64	0.025
A3. PD patients should be assessed for cognitive function using applicable neuropsychological assessment scales.	3.68 ± 0.60	3.54 ± 0.71	0.041
A4. Rehabilitation interventions for cognitive impairment should be provided to PD patients if they subjectively perceive cognitive function decline, even when neuropsychological tests show they have a normal cognitive function.	3.60 ± 0.68	3.47 ± 0.77	0.054
A5. Long-term management and follow-up are needed for PD patients with cognitive impairment.	3.79 ± 0.50	3.67 ± 0.60	0.008
A6. Early rehabilitation interventions for PD patients are beneficial in preventing cognitive impairment.	3.72 ± 0.57	3.58 ± 0.69	0.021
A7. Some physicians lack awareness and attention to cognitive impairment in PD and its rehabilitation interventions.	3.42 ± 0.72	3.07 ± 1.05	< 0.001
A8. Some nurses lack awareness and attention to cognitive impairment in PD and its rehabilitation interventions.	3.39 ± 0.74	3.04 ± 1.05	0.001

The mean practice score was 21.07 ± 8.03 (total score: 0–28, 75.25%) in the physicians and 25.27 ± 8.96 (78.97%) in the nurses, indicating active practice. The practice scores varied among nurses with different working in different departments (*P* = 0.009) (Table 2). More than 60% would educate patients to take their medications as prescribed by their physicians and advise on dietary precautions. Physicians had higher scores than the nurses for item P7 (*P* = 0.036) (Table 5).

**Table 5:** Clinical practice of physician and nurse

Practice	Physicians, n (%)	Nurses, n (%)	***P***
	Mean ± SD	Mean ± SD	
P1-1 (Physician) Develop a follow-up plan for the patient and inform them and their family of the importance of long-term follow-up. P1-2 (Nurse) Follow up patients and inform them and their families of the importance of long-term follow-up.	3.42 ± 0.74	3.41 ± 0.77	0.681
P2. Assess risk factors of cognitive impairment for all PD patients.	3.25 ± 0.80	3.22 ± 0.91	0.749
P3. Educate patients and their families about the importance of rehabilitation training.	3.49 ± 0.72	3.45 ± 0.76	0.553
P4. Educate patients and families on ways to exercise cognitive function.	3.33 ± 0.82	3.31 ± 0.84	0.415
P5. Educate patients to take their medications as prescribed by the doctor and advise on dietary precautions.	3.54 ± 0.64	3.60 ± 0.67	0.504
P6. Apply appropriate scales to assess the patient’s cognitive function.	3.17 ± 0.87	3.08 ± 1.01	0.354
P7. Be proactive in following research advances related to cognitive impairment in PD.	3.19 ± 0.88	3.03 ± 0.97	0.036

**Fig. 1:**
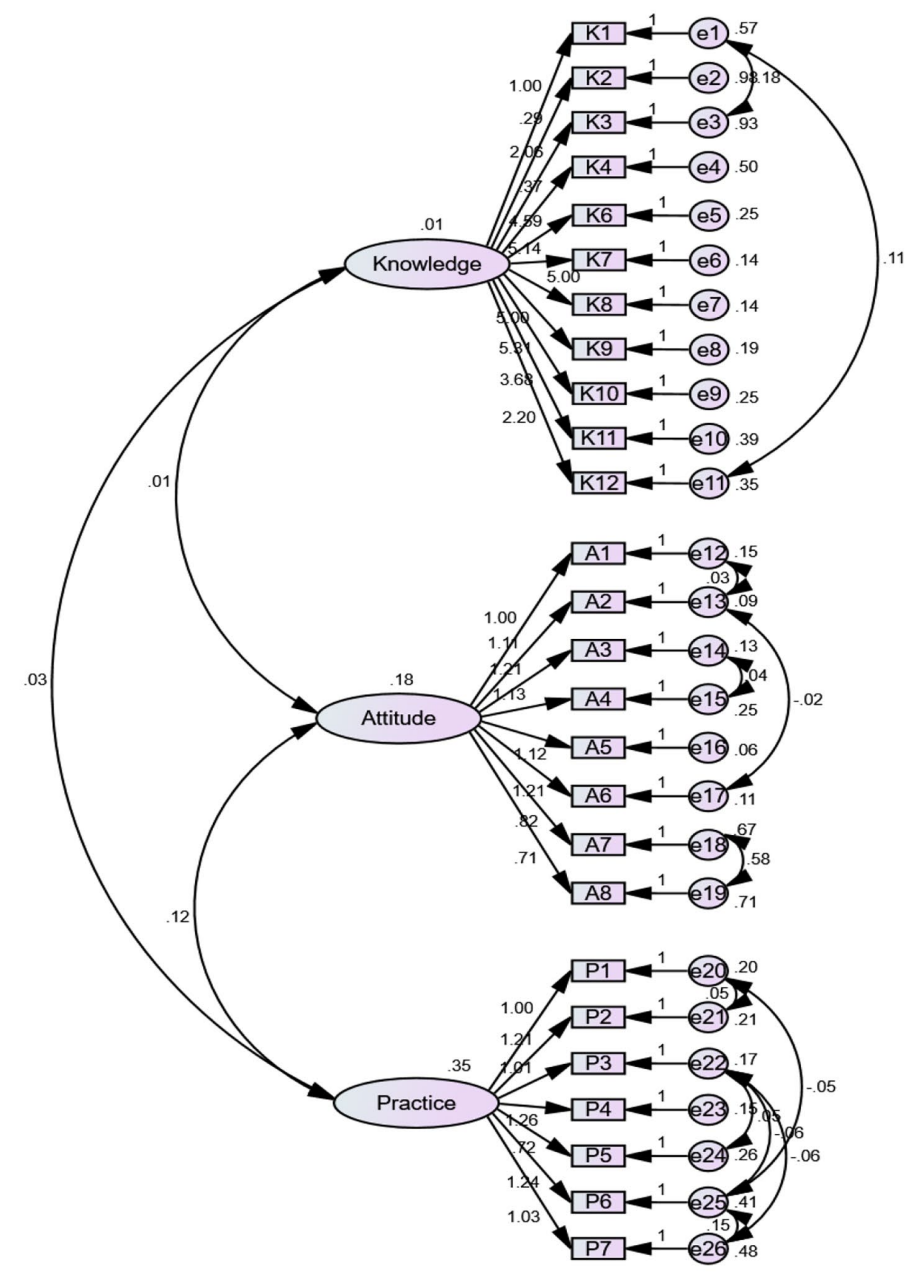
The confirmatory factor analysis (CFA) based on the KAP model

The knowledge scores were positively correlated with the attitude and practice (physician: r = 0.38, nurse: r = 0.73, both *P* < 0.001) scores, and the attitude scores positively correlated with the practice scores (physician: r = 0.44, nurse: r = 0.84, both *P* < 0.001) (Table 6). Among physicians, only the knowledge scores (OR = 4.23, 95%CI: 2.36–7.58, *P* < 0.001) and attitude scores (OR = 3.00, 95%CI: 1.67–5.37, *P* < 0.001) were independently associated with practice. Among nurses, the knowledge scores (OR = 4.31, 95%CI: 2.31–8.05, *P* < 0.001), attitude scores (OR = 5.18, 95%CI: 2.82–9.53, *P* < 0.001), and the working department (Ref: rehabilitation; neurology: OR = 2.26, 95%CI: 1.01–5.08, *P* = 0.048; public health service/chronic disease follow-up center: OR = 2.98, 95%CI: 1.12–7.92, *P* = 0.028) were independently associated with practice (Table 7). The CFA in the final study population showed that the questionnaire fits the KAP model well (Table 8; Fig. 1).

**Table 6:** Correlations among knowledge, attitude, and practice

		Knowledge	Attitude	Practice
Physician	Knowledge	1		
	Attitude	0.30 (*P* < 0.001)	1	
	Practice	0.38 (*P* < 0.001)	0.44 (*P* < 0.001)	1
Nurse	Knowledge	1		
	Attitude	0.67 (*P* < 0.001)	1	
	Practice	0.73 (*P* < 0.001)	0.84 (*P* < 0.001)	1

**Table 7:** Multivariable logistic regression analysis of practice

Participants	Variables	OR (95%CI)	***P***
**Physicians**	Knowledge score	4.23 (2.36, 7.58)	< 0.001
	Attitude score	3.00 (1.67, 5.37)	< 0.001
**Nurses**	Knowledge score	4.31 (2.31, 8.05)	< 0.001
	Attitude score	5.18 (2.82, 9.53)	< 0.001
	Department		
	Rehabilitation	Ref.	
	Neurology	2.26 (1.01, 5.08)	0.048
	Public health service/chronic disease follow-up center	2.98 (1.12, 7.92)	0.028

**Table 8:** Fitting of the CFA analysis

Indicator	Reference	Value
CMIN/DF	1–3 is excellent, 3–5 is good	2.408
RMSEA	< 0.08 is good	0.048
IFI	> 0.8 is good	0.954
TLI	> 0.8 is good	0.947
CFI	> 0.8 is good	0.953

## Discussion

This study investigated the KAP of healthcare professionals regarding cognitive dysfunction in PD and cognitive rehabilitation. The results suggested that physicians and nurses in Jiangsu have insufficient knowledge, favorable attitudes, and active practice toward cognitive impairment and cognitive rehabilitation in PD. The results might help design training and educational activities to improve the KAP of healthcare professionals toward cognitive dysfunction and cognitive rehabilitation for patients with PD.

Few studies examined the KAP of healthcare providers toward cognitive dysfunction and cognitive rehabilitation in PD. Lim et al. [18] identified key patterns in the management practices and styles of non-neurologists and gaps in knowledge and practice regarding PD management. Alcalay et al. [19] reported low knowledge and practice barriers to genetic counseling and testing in PD. Knowledge is essential to the correct management of a disease. The present study identified insufficient knowledge in physicians and nurses regarding cognitive dysfunction and cognitive rehabilitation in PD, irrespective of the department they were working in. Still, nurses working in the neurology department exhibited higher (yet insufficient) knowledge compared with the nurses from the other departments, probably because they are more exposed to knowledge transfer from the neurologists. In addition, poor knowledge among healthcare providers would result in an inability to transmit adequate knowledge to the patients and their caregivers, as shown by previous studies [24–28].

This study showed that the correct rate of most knowledge items was below 60%, indicating insufficient knowledge. Therefore, at least all knowledge items in the present study might be included in future teaching, training, and education activities to improve the KAP toward cognitive dysfunction in PD. A recent study indicated that interprofessional education could increase knowledge, promote team building, and change practice in the care of PD [29], highlighting that the different healthcare professionals have different perspectives on PD and could learn from each other.

Of note, the correlations among the three dimensions were relatively low in physicians (all r < 0.50), while the correlations among the three dimensions were relatively high in nurses (all r > 0.65). The physicians showed higher attitude and practice scores than the nurses for some items. This study was not designed to determine the reasons for these differences, but it might be because physicians’ practice was not only based on their knowledge and attitudes but also influenced by the guidelines and instructions from their supervising physicians. In addition, the training of physicians is longer than for nurses and is more focused on available treatments and disease management, while the training of nurses is more focused on performing care and nursing procedures. Future studies might be designed to examine such differences.

This study had several limitations. The participants were from Jiangsu only, resulting in a relatively small sample size and limiting the generalizability of the results. Indeed, generalizability was always an issue with KAP surveys since such surveys only assess a specific population at a specific time [16, 17]. Still, the same questionnaire could be used in the future to determine whether the KAP evolves in time or to determine the efficacy of future training and continuous education activities. Almost all nurses were female, which could influence the results. In addition, KAP surveys were biased by local practice since the investigators tended to design the items according to their local practice and experience. Finally, all KAP studies are limited by the social desirability bias. It is an inherent bias in all KAP studies, and there is a possibility that some participants answered what they know they should do instead of what they are doing [30, 31].

## Conclusions

The physicians and nurses have insufficient knowledge, favorable attitudes, and active practice toward cognitive dysfunction in PD and cognitive rehabilitation. This study identified gaps in KAP and suggested educational activities, such as training and competition, to improve the KAP towards cognitive dysfunction in PD.

### Electronic supplementary material

Below is the link to the electronic supplementary material.

Supplementary Material 1

## Data Availability

All data generated or analysed during this study are included in this published article.

## References

[1] Kalia LV, Lang AE (2015). Parkinson’s Disease. Lancet.

[2] Homayoun H (2018). Parkinson Disease. Ann Intern Med.

[3] Benazzouz A, Mamad O, Abedi P, Bouali-Benazzouz R, Chetrit J (2014). Involvement of dopamine loss in extrastriatal basal ganglia nuclei in the pathophysiology of Parkinson’s Disease. Front Aging Neurosci.

[4] Aosaki T, Miura M, Suzuki T, Nishimura K, Masuda M (2010). Acetylcholine-dopamine balance hypothesis in the striatum: an update. Geriatr Gerontol Int.

[5] Lees AJ, Hardy J, Revesz T (2009). Parkinson’s Disease. Lancet.

[6] Kasten M, Kertelge L, Bruggemann N, van der Vegt J, Schmidt A, Tadic V, Buhmann C, Steinlechner S, Behrens MI, Ramirez A (2010). Nonmotor symptoms in genetic Parkinson Disease. Arch Neurol.

[7] Todorova A, Jenner P, Ray Chaudhuri K (2014). Non-motor Parkinson’s: integral to motor Parkinson’s, yet often neglected. Pract Neurol.

[8] Buter TC, van den Hout A, Matthews FE, Larsen JP, Brayne C, Aarsland D (2008). Dementia and survival in Parkinson Disease: a 12-year population study. Neurology.

[9] Goodarzi Z, Mrklas KJ, Roberts DJ, Jette N, Pringsheim T, Holroyd-Leduc J (2016). Detecting depression in Parkinson Disease: a systematic review and meta-analysis. Neurology.

[10] Broen MP, Narayen NE, Kuijf ML, Dissanayaka NN, Leentjens AF (2016). Prevalence of anxiety in Parkinson’s Disease: a systematic review and meta-analysis. Mov Disord.

[11] Hong CT, Tan S, Huang TW (2021). Psychotherapy for the treatment of anxiety and depression in patients with Parkinson Disease: a Meta-analysis of Randomized controlled trials. J Am Med Dir Assoc.

[12] Seppi K, Ray Chaudhuri K, Coelho M, Fox SH, Katzenschlager R, Perez Lloret S, Weintraub D, Sampaio C (2019). The collaborators of the Parkinson’s Disease Update on Non-motor symptoms Study Group on behalf of the Movement Disorders Society Evidence-Based Medicine C: update on treatments for nonmotor symptoms of Parkinson’s disease-an evidence-based medicine review. Mov Disord.

[13] Diez-Cirarda M, Ibarretxe-Bilbao N, Pena J, Ojeda N (2018). Efficacy of cognitive rehabilitation in Parkinson’s Disease. Neural Regen Res.

[14] Alzahrani H, Venneri A (2018). Cognitive Rehabilitation in Parkinson’s Disease: a systematic review. J Parkinsons Dis.

[15] Guglietti B, Hobbs D, Collins-Praino LE (2021). Optimizing cognitive training for the treatment of cognitive dysfunction in Parkinson’s Disease: current limitations and future directions. Front Aging Neurosci.

[16] Andrade C, Menon V, Ameen S, Kumar Praharaj S (2020). Designing and conducting knowledge, attitude, and practice surveys in Psychiatry: practical Guidance. Indian J Psychol Med.

[17] World Health Organization. : *Advocacy, communication and social mobilization for TB control: a guide to developing knowledge, attitude and practice surveys* http://whqlibdoc.who.int/publications/2008/9789241596176_eng.pdf. Accessed November 22; 2022.

[18] Lim I, Saffari SE, Neo S (2022). A cross-sectional study of knowledge and practices in the management of patients with Parkinson’s Disease amongst public practice-based general practitioners and geriatricians. BMC Health Serv Res.

[19] Alcalay RN, Kehoe C, Shorr E, Battista R, Hall A, Simuni T, Marder K, Wills AM, Naito A, Beck JC (2020). Genetic testing for Parkinson Disease: current practice, knowledge, and attitudes among US and Canadian movement disorders specialists. Genet Med.

[20] Poewe W, Gauthier S, Aarsland D, Leverenz JB, Barone P, Weintraub D, Tolosa E, Dubois B (2008). Diagnosis and management of Parkinson’s Disease Dementia. Int J Clin Pract.

[21] Xiong M, Wang L, Yu HL, Han H, Mao D, Chen J, Zeng Y, He N, Liu ZG, Wang ZY (2016). Ginkgetin exerts growth inhibitory and apoptotic effects on osteosarcoma cells through inhibition of STAT3 and activation of caspase-3/9. Oncol Rep.

[22] Wang Lijuan F, Shujun, Kun N. Diagnosis and treatment guidelines for mild cognitive impairment in Parkinson’s Disease in China (2020 edition)[J]. Chin J Neuropsychiatric Disorders 2021, 47.

[23] Litvan I, Goldman JG, Troster AI, Schmand BA, Weintraub D, Petersen RC, Mollenhauer B, Adler CH, Marder K, Williams-Gray CH (2012). Diagnostic criteria for mild cognitive impairment in Parkinson’s Disease: Movement Disorder Society Task Force guidelines. Mov Disord.

[24] Pan S, Stutzbach J, Reichwein S, Lee BK, Dahodwala N (2014). Knowledge and attitudes about Parkinson’s Disease among a diverse group of older adults. J Cross Cult Gerontol.

[25] Kaddumukasa M, Kakooza A, Kaddumukasa MN, Ddumba E, Mugenyi L, Sajatovic M, Katabira E (2015). Knowledge and attitudes of Parkinson’s Disease in Rural and Urban Mukono District, Uganda: a cross-sectional, community-based study. Parkinsons Dis.

[26] Sadowski CA, Jones CA, Gordon B, Feeny DH (2007). Knowledge of risk factors for falling reported by patients with Parkinson Disease. J Neurosci Nurs.

[27] Youn J, Oh E, Park J, Park S, Kim JS, Jang W (2016). Public Awareness and Knowledge about Parkinson’s Disease: a National Population Based Survey in South Korea. Neuroepidemiology.

[28] Jitkritsadakul O, Boonrod N, Bhidayasiri R (2017). Knowledge, attitudes and perceptions of Parkinson’s Disease: a cross-sectional survey of Asian patients. J Neurol Sci.

[29] Cohen EV, Hagestuen R, Gonzalez-Ramos G, Cohen HW, Bassich C, Book E, Bradley KP, Carter JH, Di Minno M, Gardner J (2016). Interprofessional education increases knowledge, promotes team building, and changes practice in the care of Parkinson’s Disease. Parkinsonism Relat Disord.

[30] Bergen N, Labonte R (2020). Everything is perfect, and we have no problems: detecting and limiting Social Desirability Bias in qualitative research. Qual Health Res.

[31] Latkin CA, Edwards C, Davey-Rothwell MA, Tobin KE (2017). The relationship between social desirability bias and self-reports of health, substance use, and social network factors among urban substance users in Baltimore, Maryland. Addict Behav.

