# Supplementary material for: Knowledge, attitude, and practice of healthcare professionals toward cognitive dysfunction in Parkinson’s disease and cognitive rehabilitation
Source: BMC Med Educ. 2024 Jan 4;24:26. doi: 10.1186/s12909-023-04989-5 (PMC10768106; doi:10.1186/s12909-023-04989-5)
Supplement: Supplementary file 1 — Supplementary Material 1 [file 12909_2023_4989_MOESM1_ESM.docx]

| Questionnaire No. : | | | |
| --- | --- | --- | --- |
| Dear healthcare professional:  We are researchers at the hospital, and we would like to invite you to participate in our study. In order to provide a basis for developing scientific intervention strategies that may help more people and improve their health in the future, this study aims to understand the knowledge, attitudes, and practices of healthcare professionals regarding cognitive dysfunction in Parkinson’s disease (PD) and rehabilitation interventions. Your participation in this study is voluntary, and this study has been approved by the Medical Ethics Committee of the hospital. If you agree to participate in this study, please refer to the following instructions.  1. Please complete the questionnaire. There is no absolute right or wrong answer. You just need to fill in according to your actual situation. You can ask us any questions during the answering process, and when you are finished, please submit it in time.  2. This study is a simple questionnaire; it will not cause harm to your physical and psychological condition, but it may involve some private questions, such as your gender, age, etc. We will keep it strictly confidential and will not disclose your information. Please feel free to fill it out.  3. As a participant, you can stay informed about information and progress related to this study. If you decide to withdraw from it, please inform us that your data will not be included in the results.  Finally, we sincerely thank you for taking the time out of your busy schedule to support our scientific study!  □ I have knowledge of and consent to the use of the collected data for scientific study.  Informed Consent Signature：  Participation date： Year Month Day | | | |
| **Part I** **Basic Information** | | | |
| **1. Your gender：** | | a. Male | b. Female |
| **2. Your age： years old.** | | | |
| **3. Your education：** | | a. High School/Technical secondary school  b. Junior college/Bachelor’s degree  c. Bachelor’s degree  d. PhD degree | |
| **4. The nature of the institution you work in:** | | a. Public Primary Hospitals  b. Public Secondary Hospitals  c. Public Tertiary Hospitals  d. Private Hospitals | |
| **5. Your occupation：** | | a. Physician  b. Nurse | |
| **6. Your department：** | | a. Neurology  b. Rehabilitation  c. Public Health Service/Chronic Disease Follow-up Center (or other departments with chronic disease follow-up functions) | |
| **7. Your professional title：** | | a. None  b. Primary title  c. Middle title  d. Vice-senior Title  e. Senior Title | |
| **8. Your years of work：** | | a. <1 year  b. ≥1 but <3 years  c. ≥3 but <5 years  d. ≥5 but <10 years  e. ≥10 years | |

**Part II Awareness regarding cognitive dysfunction in PD and its rehabilitation interventions**

| **1. Cognitive dysfunction is one of the common non-motor symptoms of Parkinson’s disease (PD), including____________________. (Multiple choice)** | a. Mild cognitive impairment(MCI) in PD  b. Moderate cognitive impairment in PD  c. Parkinson’s disease dementia (PDD)  d. Unknown | | |
| --- | --- | --- | --- |
| **2. MCI is an intermediate state between normal cognitive function and PDD, it progressing over time to_______. (Single choice)** | a. One-way progress toward PDD  b. Vesting to normal cognitive function or developing into PDD  c. Unknown | | |
| **3. The risk factors for the development of dementia in PD patients include_______. (Multiple choice)** | a. At the age of 75 years or older  b. Low educational level  c. Have a disease duration of more than 10 years  d. With MCI  e. Have rapid eye movement sleep behavior disorder  f. Unknown | | |
| **4. The medications that should be discontinued in PD patients with Cognitive dysfunction include：_______. (Multiple choice)** | a. Rivastigmine  b. Benzhexol  c. Amantadine  d. Unknown | | |
| **5. MCI in PD does not appear earlier than motor impairment in PD; is this statement correct?** | a. Correct | b. Wrong | c. Unknown |
| **6. The Montreal Cognitive Assessment (MoCA), the Parkinson Disease Cognitive Rating Scale (PD-CRS), and the Mattis Dementia Rating Scale-2 (MDRS-2) are the three scales currently recommended for evaluating overall cognitive function in PD with better validity and reliability; do you know about it?** | a. Know | b. Know a little | c. Unknown |
| **7. The rehabilitation treatments for PD that have proven effective abroad include physical and motor therapy, occupational therapy, speech-language therapy, and swallowing therapy; do you know about it?** | a. Know | b. Know a little | c. Unknown |
| **8. PD patients with cognitive impairment can undergo cognitive rehabilitation training, such as memory training, attention training, etc; do you know how to do cognitive training?** | a. Know | b. Know a little | c. Unknown |
| **9. Aerobic exercise may be effective in improving executive function in PD patients, and appropriate aerobic exercise, such as horizontal exercise bicycles, is recommended for PD patients with MCI; do you know about it?** | a. Know | b. Know a little | c. Unknown |
| **10. Transcranial direct current stimulation (tDCS) treatment may be considered for PD patients with MCI; do you know about it?** | a. Know | b. Know a little | c. Unknown |
| **11. Do you know the dietary precautions for PD patients? such as not eating high-protein foods when taking levodopa preparations for control?** | a. Know | b. Know a little | c. Unknown |
| **12. MCI in PD can appear in the early stage of the disease and may even precede the onset of motor impairment; is this statement correct?** | a. Correct | b. Wrong | c. Unknown |

| **Part III Attitude regarding cognitive dysfunction in PD and its rehabilitation interventions** | | | | | | |
| --- | --- | --- | --- | --- | --- | --- |
| 1. **You think that early diagnosis and intervention of MCI in PD are of clinical importance.** | a. Strongly agree | b. Agree | c. Neutral | d. Disagree | e. Strongly disagree | |
| **2. You think that there is no absolute fixed model for the treatment of PD and that an individualized rehabilitation intervention program needs to be developed based on the patient’s condition.** | a. Strongly agree | b. Agree | c. Neutral | d. Disagree | e. Strongly disagree | |
| **3. You think PD patients should be assessed for cognitive function using applicable neuropsychological assessment scales.** | a. Strongly agree | b. Agree | c. Neutral | d. Disagree | e. Strongly disagree | |
| **4. You think that rehabilitation interventions for cognitive impairment should be provided to PD patients if they subjectively perceive cognitive function decline, even when neuropsychological test results show they have normal cognitive function.** | a. Strongly agree | b. Agree | c. Neutral | d. Disagree | e. Strongly disagree | |
| **5. You think that long-term management and follow-up are needed for PD patients with cognitive impairment.** | a. Strongly agree | b. Agree | c. Neutral | d. Disagree | e. Strongly disagree | |
| **6. You think that early rehabilitation interventions for PD patients are beneficial in preventing the onset of cognitive impairment.** | a. Strongly agree | b. Agree | c. Neutral | d. Disagree | e. Strongly disagree | |
| **7. You think that in current clinical practice, some physicians lack awareness and attention to cognitive impairment in PD and its rehabilitation interventions.** | a. Strongly agree | b. Agree | c. Neutral | d. Disagree | e. Strongly disagree | |
| **8. You think that in current clinical practice, some nurses lack awareness and attention to cognitive impairment in PD and its rehabilitation interventions.** | a. Strongly agree | b. Agree | c. Neutral | d. Disagree | e. Strongly disagree | |
| **Part IV Practice regarding cognitive dysfunction in PD and its rehabilitation interventions** | | | | | |  |
| **1-1 (Physicians) You will develop a follow-up plan for the patient and inform the patient and their family of the importance of long-term follow-up.** | a. Very consistent with | b. Consistent with | c. Neutral | d. Inconsistent with | e. Very inconsistent with |  |
| **1-2 (Nurses) According to the follow-up plan developed by the doctor, you will follow up with patients for advocacy and education and inform them and their families of the importance of long-term follow-up.** |  |  |  |  |  |  |
| **2. For all PD patients, you will assess risk factors for the progression of their cognitive impairment.** | a. Very consistent with | b. Consistent with | c. Neutral | d. Inconsistent with | e. Very inconsistent with |  |
| **3. You will educate patients and their families about the importance of rehabilitation training.** | a. Very consistent with | b. Consistent with | c. Neutral | d. Inconsistent with | e. Very inconsistent with |  |
| **4. You will educate patients and families on ways to exercise cognitive function, such as you will instruct patients on how to perform memory training, executive function training, etc.** | a. Very consistent with | b. Consistent with | c. Neutral | d. Inconsistent with | e. Very inconsistent with |  |
| **5. You will educate patients to take their medications as prescribed by the doctor and advise on dietary precautions.** | a. Very consistent with | b. Consistent with | c. Neutral | d. Inconsistent with | e. Very inconsistent with |  |
| **6. You will be able to select and use appropriate scales for assessing your patient’s cognitive function, such as PD⁃CRS, MoCA, MDRS-2, Scales for Outcomes in Parkinson’s Disease-Cognition (SCOPA-Cog), and Mini-Mental State Examination (MMSE).** | a. Very consistent with | b. Consistent with | c. Neutral | d. Inconsistent with | e. Very inconsistent with |  |
| **7. You will be proactive in following research advances related to cognitive impairment in PD.** | a. Very consistent with | b. Consistent with | c. Neutral | d. Inconsistent with | e. Very inconsistent with |  |
